# Accelerating development of high-risk neuroblastoma patient-derived xenograft models for preclinical testing and personalised therapy

**DOI:** 10.1038/s41416-019-0682-4

**Published:** 2020-01-10

**Authors:** Alvin Kamili, Andrew J. Gifford, Nancy Li, Chelsea Mayoh, Shu-Oi Chow, Timothy W. Failes, Georgina L. Eden, Roxanne Cadiz, Jinhan Xie, Robyn E. Lukeis, Murray D. Norris, Michelle Haber, Geoffrey B. McCowage, Greg M. Arndt, Toby N. Trahair, Jamie I. Fletcher

**Affiliations:** 1 Children’s Cancer Institute, Lowy Cancer Research Centre, UNSW Sydney, Sydney, NSW Australia; 2 School of Women’s and Children’s Health, UNSW Sydney, Sydney, NSW Australia; 3 Department of Anatomical Pathology, Prince of Wales Hospital, Randwick, NSW Australia; 4 School of Medical Sciences, UNSW Sydney, Sydney, NSW Australia; 5 ACRF Drug Discovery Centre for Childhood Cancer, Children’s Cancer Institute, Lowy Cancer Research Centre, UNSW Sydney, Sydney, NSW Australia; 6 Cytogenetics Laboratory, SydPath, St Vincent’s Hospital, Darlinghurst, NSW Australia; 7 University of New South Wales Centre for Childhood Cancer Research, UNSW Sydney, Sydney, NSW Australia; 8 Cancer Centre for Children, Children’s Hospital at Westmead, Westmead, NSW Australia; 9 Kids Cancer Centre, Sydney Children’s Hospital, Randwick, NSW Australia

**Keywords:** Paediatric cancer, Cancer models

## Abstract

**Background:**

Predictive preclinical models play an important role in the assessment of new treatment strategies and as avatar models for personalised medicine; however, reliable and timely model generation is challenging. We investigated the feasibility of establishing patient-derived xenograft (PDX) models of high-risk neuroblastoma from a range of tumour-bearing patient materials and assessed approaches to improve engraftment efficiency.

**Methods:**

PDX model development was attempted in NSG mice by using tumour materials from 12 patients, including primary and metastatic solid tumour samples, bone marrow, pleural fluid and residual cells from cytogenetic analysis. Subcutaneous, intramuscular and orthotopic engraftment were directly compared for three patients.

**Results:**

PDX models were established for 44% (4/9) of patients at diagnosis and 100% (5/5) at relapse. In one case, attempted engraftment from pleural fluid resulted in an EBV-associated atypical lymphoid proliferation. Xenogeneic graft versus host disease was observed with attempted engraftment from lymph node and bone marrow tumour samples but could be prevented by T-cell depletion. Orthotopic engraftment was more efficient than subcutaneous or intramuscular engraftment.

**Conclusions:**

High-risk neuroblastoma PDX models can be reliably established from diverse sample types. Orthotopic implantation allows more rapid model development, increasing the likelihood of developing an avatar model within a clinically useful timeframe.

## Background

Neuroblastoma is the most common extracranial solid tumour of children and accounts for 15% of all paediatric cancer deaths.^1,2^ The cure rates of children with high-risk neuroblastoma remain less than 50%^3^ and as low as 10% for relapsed and refractory disease.^4^ Treatment-related acute toxicities and the high prevalence of significant late effects also present major challenges.^5,6^ Realistic laboratory models that reflect the genetic diversity of high-risk neuroblastoma have an important role to play in preclinical assessment of new agents or combinations, in validating the relevance of mutations as actionable targets and in modelling the development of drug resistance.^7,8^ For the individual patient, personalised or avatar models are playing an increasingly important role in personalised oncology.^9,10^

Patient-derived xenograft (PDX) models are generated from the implantation of patient tumour sample directly into immunodeficient mice, and the subsequent passage of tumour material from animal to animal.^11^ Substantial evidence from diverse solid and haematopoietic tumours suggests that PDX models may be more informative preclinical models than cell line xenografts,^12,13^ and more accurately represent the histopathologic and molecular features of the original tumour, having avoided the in vitro adaptation that inevitably results from long-term growth and expansion of tumour cells in tissue culture conditions.^11,14–17^ Personalised PDX models can allow more comprehensive supporting data for precision medicine by expanding limited patient material for additional analyses, such as unbiased ex vivo drug screening and by providing an in vivo platform for evidence-based validation and prioritisation of therapeutic options. However, limited engraftment success rates, lengthy model establishment time and high maintenance costs all pose major challenges to the routine use of PDX models for personalised therapy.^11,18^ Optimising the methodology to improve engraftment efficiency is critical for meeting urgent clinical needs in the era of personalised medicine.

Establishment of neuroblastoma PDX models from primary tumour samples was first reported over 25 years ago^19–21^ and has been described in more recent accounts.^22–28^ Herein, we demonstrate the feasibility of developing high-risk neuroblastoma PDX models at a high success rate from multiple sources of tumour-bearing patient materials at both diagnosis and relapse and from primary and metastatic tumour sites, and for the first time, residual tumour cells from cytogenetic analysis. We also show that the engraftment time is influenced not only by the number of tumour cells inoculated but also by the site of inoculation, with orthotopic (adrenal) engraftment allowing more rapid model generation than subcutaneous engraftment. Finally, we also describe some of the challenges in developing PDX models—the development of xenogeneic graft versus host disease in recipient mice and the proliferation of Epstein–Barr virus (EBV)-infected cells rather than tumour cells—and provide strategies for obviating these hurdles.

## Methods

### Patient samples and processing

Fresh tumour specimens were obtained from patients at the Sydney Children’s Hospital Network (SCHN) under approval by the SCHN Human Research Ethics Committee (LNR/14/SCHN/392, LNR/14/SCHN/497). The primary purpose for developing the PDX models was to establish and improve xenografting approaches, to develop models to enable future preclinical testing, including in the context of personalised medicine. The relapse samples were from patients enrolled in a pilot feasibility study (TARGET) for a subsequent personalised medicine clinical trial (PRISM; ClinicalTrials.gov Identifier NCT03336931), however without intention to return data to the treating clinician. Informed parental or guardian consent was obtained for each patient. The initial diagnosis of neuroblastoma was confirmed for each patient by a consultant paediatric pathologist. Samples were collected and transported in L-15 medium (Life Technologies, Carlsbad, CA, USA) at room temperature within 2–8 h after surgery. Solid tumour sample dissociation was performed enzymatically using a human tumour dissociation kit and a GentleMACS dissociator (Miltenyi Biotec, Bergisch Gladbach, Germany) into a single-cell suspension. Smaller samples were engrafted as 2–5-mm fragments. Bone marrow samples were collected in EDTA tubes and mononuclear cells isolated by Ficoll separation. Depletion of T lymphocytes was performed with human CD3 microbeads and an autoMACS Pro (Miltenyi Biotec) prior to assessment by flow cytometry and the CD3-negative population used for inoculation. Cells from pleural fluid were isolated by centrifugation and washed with red blood cell lysis buffer prior to inoculation. Cells from cytogenetics culture were obtained after completion of diagnostic analyses, harvested in EDTA-trypsin and washed in PBS prior to inoculation.

### Establishment of PDX models

All animal studies were performed in accordance with the guidelines approved by the University of New South Wales Animal Care and Ethics Committee (ACEC 17/101B) and the requirements of the Australian Code for the Care and Use of Animals for Scientific Purposes. Animals were housed in a specific pathogen-free environment in Techniplast research animal cages with filter tops and floor area of 400 cm^2^ with 2–6 mice/cage, and under 12:12 light/dark cycles. Bedding, enviro dry and igloos were provided for environmental enrichment. Irradiated rat and mouse breeder cubes and water were provided ad libitum. All animal procedures were performed inside a biosafety cabinet under sterile conditions during the animal’s light time cycle. Dissociated tumour cells, bone marrow, pleural fluid and cytogenetic cells (0.1–10 million cells) were mixed in RPMI (Life Technologies) and growth factor-reduced Matrigel (Corning Inc., Corning, NY, USA) at equal volume ratio and implanted subcutaneously using a 27-gauge needle (Terumo, Tokyo, Japan) into the flank of female NOD.Cg-*Prkdc*^*scid*^ *Il2rg*^*tm1Wjl*^/SzJ (NSG) mice^29^ obtained from Australian Bioresources (Moss Vale, NSW, Australia). For implantation of tumour fragments, mice were anaesthetised using a Stinger anaesthetic machine (DarvallVet, Gladesville, NSW, Australia) with 2–2.5% isoflurane (Isothesia, Henry Schein, Melville, NY, USA) and 800 mL/min oxygen. A 5-mm horizontal incision was made on the dorsum of the mice, 10 mm rostral to the base of the tail, to create a subcutaneous pocket. A 2–5-mm fragment of tumour dipped in growth factor-reduced Matrigel was inserted and the incision site was closed with a wound clip. Buprenorphine (0.1 mg/kg, intraperitoneal) (Provet, Castle Hill, Australia) was administered for analgesia. Tumour growth was measured by Vernier callipers at least twice a week. Mice were euthanised once tumour size reached 1000 mm^3^, calculated using the formula: Length × Width × Height/2, by CO_2_ overdose followed by cervical dislocation. Tumours were triaged for histological and molecular analyses, cryopreservation and expansion in secondary recipient mice. Failure to engraft was defined as no measurable tumour at maximum holding time (12 months) and was confirmed by necropsy.

### Engraftment site comparison

For each of three patients, a single tumour sample was dissociated and inoculated simultaneously into randomised cohorts of NSG mice at subcutaneous, intramuscular or orthotopic sites. A fixed cell number per mouse was used for each inoculation based on sample availability (5 × 10^5^, 1 × 10^5^ and 1.35 × 10^5^ cells per mouse for A6580, A6912 and A7560, respectively). Subcutaneous inoculation, as well as anaesthesia and analgesia for intramuscular and orthotopic engraftment were performed as described above. For intramuscular engraftment, a 10-mm midline dorsal skin incision was made, 15 mm rostral to the base of the tail.^30^ Cells suspended in a mixture of 10 μL of RPMI and 10 μL of Matrigel were injected into the paraspinal muscle and the incision closed with 9-mm wound clips (Fine Scientific Tools, North Vancouver, BC, Canada). Orthotopic (adrenal) engraftment was performed as previously described.^31^ Briefly, a 15-mm skin incision was made adjacent to the caudal border of the spleen and the superior tip of the left kidney, followed by a 10-mm incision into the abdominal wall to expose the left adrenal gland. Cells suspended in a mixture of 10 μL of RPMI and 10 μL of Matrigel were injected into the fat pad surrounding the left adrenal gland. The abdominal wall was closed with a polyglactin-coated Vilet absorbable suture (Riverpoint Medical, Portland, OR, USA) and the skin incision was closed with wound clips. Mice were monitored daily for 7 days post surgery, and wound clips removed on day 7 post surgery. Intramuscular engraftment was assessed as described above. Orthotopic tumours were assessed by weekly ultrasound imaging and with callipers upon killing to confirm imaging-based measurement. Mice were euthanised by CO_2_ overdose followed by cervical dislocation. Event-free survival (EFS) was determined as described previously.^32^

### Ultrasound imaging

Mice were depilated (~10 cm^2^) adjacent to the left adrenal gland and prewarmed Aquasonic Clear Ultrasound Transmission Gel (Parker Laboratories, Fairfield, NJ, USA) was applied to the area. Imaging was performed using VisualSonics Vevo 2100 (VisualSonics, Toronto, Canada). A linear-array transducer (MS-250) traversed the selected area, acquiring 2D images at ~100-μm intervals, which were reconstructed as a 3D image in a dynamic cube format and analysed using Vevo Lab 1.7.0 software (VisualSonics). Tumour boundaries were manually outlined in parallel slices within the 3D image and the final tumour volume calculated as the sum of the outlined tumour boundary for each slice.

### Histology and immunohistochemistry

Tumours and organs were fixed in formalin, embedded in paraffin and then sectioned and stained for histopathologic assessment by a practicing paediatric pathologist (AJG). Stains included haematoxylin and eosin (H&E), Mason trichrome, orcein elastic and Papanicolaou and Giemsa. Immunohistochemical (IHC) staining included NB84 (1:400, Leica, Nussloch, Germany), synaptophysin (1:200, Leica), CD56 (1:100, Leica), PHOX2B (1:1000, Abcam, Cambridge, UK), CD45 (1:400, Leica), CD20 (1:200, Leica) and CD3 (1:200, Leica). For EBV-encoded RNA in situ hybridisation (EBER-ISH), slides were stained using BOND EBER probe (Leica). Images were captured using an Olympus BX53 light microscope and CD73 camera with CellSens software.

### Copy-number analysis and short-tandem repeat (STR) profiling

DNA was extracted from patient and PDX tumours using the ReliaPrep^TM^gDNA Tissue Miniprep System (Promega, Madison, WI, USA) or Allprep DNA/RNA/Protein Mini Kit (Qiagen, Hilden, Germany). For copy-number analysis using single-nucleotide polymorphism (SNP) arrays, DNA samples were processed using the Illumina InfiniumOmni2.5–8 according to the manufacturer’s guidelines to interrogate ~2.5 M SNP loci (Australian Genome Research Facility). Standard clustering and genotyping were performed using GenomeStudio v2.0.3 (Illumina, San Diego, CA, USA) using default settings to collect SNP calls. Regions of copy-number variations (CNV) were called using cnvPartition2.0 with default parameters. B-allele frequency (BAF), log R ratio (LRR) and CNV plots were generated using package karyoploteR within the R statistical software package. CNV was determined as a function of BAF and LRR as previously described.^33^ Patient CNV data were also validated against the clinical data obtained through routine diagnostic DNA microarrays (Affymetrix Cytoscan 750K). Short-tandem repeat (STR) profiling results were analysed using PowerPlexR 18D System at the Kinghorn Centre for Clinical Genomics (Sydney, Australia).

### Flow cytometry

Infiltration of human lymphocytes into mouse liver and spleen was determined by staining with APC mouse anti-human CD45 and FITC rat anti-mouse CD45. T-lymphocyte-depleted samples were analysed using PE mouse anti-human CD3 and APC mouse anti-human CD45. B lymphocytes in PDX tumours were stained with PE mouse anti-human CD19. For each sample, red blood cells were lysed by BD FACS lysing solution. PE mouse anti-human GD2 staining was used to determine the human neuroblastoma cell content in PDX tumours. All reagents were from BD Biosciences (Franklin Lakes, NJ, USA). Data were acquired with the LSRFortessa (BD Biosciences) and analysed with FlowJo software.

### High-throughput drug screening

Dissociated patient and PDX-tumour cells were seeded on 384-well plates at 2000 cells/well in 25 μL of Iscove’s Modified Dulbecco’s Media, 20% foetal calf serum and 1× insulin–transferrin–selenium (Life Technologies). Human neuroblastoma cell content in the PDX tumour was determined by flow cytometry. After 72 h, cells were treated with a library of 165 anticancer drugs. After a further 72 h, Cell-titer glo 2D (Promega) was added to measure ATP as a surrogate for cell viability. Cell viability as a function of drug concentration was plotted with a three-parameter sigmoidal dose–response curve to determine area under the curve (AUC) values. Correlation of drug sensitivity (AUC values) in patient tumour cells compared with that for PDX-tumour cells was assessed using Pearson’s correlation analysis.

## Results

### Establishment of high-risk neuroblastoma PDX models

Tumour samples were obtained from two consecutive groups of patients: newly diagnosed high-risk neuroblastoma patients from Sydney Children’s Hospital, Randwick (nine patients) and patients at the point of relapse from either Sydney Children’s Hospital Randwick or Children’s Hospital Westmead (five patients) (Table 1). For two patients, both diagnosis and relapse samples were obtained. For three patients, multiple sample types were obtained. Samples (19 in total) included primary solid tumour, intracranial, liver, lymph node and bone marrow metastases, peripheral blood and pleural fluid, in addition to residual cells from cytogenetics analysis (Table 1). PDX models were successfully established for 4 of 9 patients (44%) at diagnosis and 5 of 5 patients (100%) at relapse. On per-sample basis, engraftment rates were 4 of 13 samples (31%) at diagnosis and 6 of 6 samples (100%) at relapse. For the ten samples that were successfully engrafted, the mean time to 1000-mm^3^ tumour was 107 days (range 33–210 days). For the remaining samples, there was no evidence of engraftment after 12 months. The established models represented a wide patient age range at the time of diagnosis (0.3–7.5 years) and were derived from both MYCN-amplified^4^ and non-amplified^6^ samples. Engraftment at diagnosis was associated with poor outcome (*P* = 0.048, Fisher’s exact test, Supplementary Table S1). Notably, in three of four cases where engraftment was successful at diagnosis, the patient subsequently relapsed or progressed and died of disease. For two of these patients, a model was also established at relapse: CCI-NB01-DMC and CCI-NB01-RMT from A6698 and CCI-NB02-DMB and CCI-NB02-RPT from A6912. Establishment of models from residual cytogenetics cultures was successful in all four cases. In two of these cases, other tumour material from the same patient inoculated in parallel failed to engraft, while in one case no other sample was available. In the single example (A6580) where a model was generated by both direct engraftment (CCI-NB06-RMT) and via cytogenetics culture (CCI-NB06-RMC), the engraftment time was similar (72 and 88 days, respectively). Each of the established models can be serially passaged and the growth time of xenografts from inoculation to 1000 mm^3^ in the secondary passage is shown in Supplementary Table S2.

**Table 1:** Characteristics of the patients for high-risk neuroblastoma PDX models.

Patient ID	Phase of therapy	Age at diagnosis (y)	INSS stage	MYCN amplification	Patient status	Tumour source	PDX ID^a^	Endpoint for initial mouse (d)	Tumour to PDX correlation (*r* value)^b^
A6698	Diagnosis	5	4	No	DOD	Skull metastasis (via cytogenetics cells)	CCI-NB01-DMC	96	0.58
A6912	Diagnosis	0.8	4	Yes	DOD	BM metastasis Intra-abdominal tumour	CCI-NB02-DMB not established	96	0.82
A7012	Diagnosis	2.8	4	Yes	DOD	Intra-abdominal tumour (via cytogenetics cells) Intra-abdominal tumour Pleural fluid	CCI-NB03-DPC not established	121	0.71
A7167	Diagnosis	3.5	4	Yes	AWD	LN metastasis (via cytogenetics cells) LN metastasis	CCI-NB04-DML not established	187	0.035
A5724	Diagnosis	0.9	4	No	AWD	BM metastasis	Not established		
A6652	Diagnosis	2.1	4	No	AWD	Left adrenal tumour	Not established		
A6744	Diagnosis	3	4	No	AWD	LN metastasis	Not established		
A7025	Diagnosis	0.3	4	No	AWD	Liver metastasis	Not established		
A7069	Diagnosis	6.5	4	Yes	AWD	Peripheral blood	Not established		
A5723	Relapse	7.5	4	No	AWD	BM metastasis	CCI-NB05-RMB	210	0.9
A6580	Relapse	4.2	4	No	AWD	Intracranial metastasis	CCI-NB06-RMT	72	0.88
						Intracranial metastasis (via cytogenetics cells)	CCI-NB06-RMC	88	0.95
A6698	Relapse	5	4	No	DOD	Intradural metastasis	CCI-NB01-RMT	76	0.91
A6912	Relapse	0.8	4	Yes	DOD	Intra-abdominal tumour	CCI-NB02-RPT	33	0.99
A7056	Relapse	2	4	No	DOD	Intracranial metastasis	CCI-NB07-RMT	91	0.84

*BM* bone marrow, *LN* lymph node, *AWD* alive with disease at the last follow-up, *DOD* died of disease, *y* years, *d* days

^a^The PDX ID consists of institutional code, disease initial and sequence number, and three letters representing xenograft sample characteristics (*D* diagnosis, *R* relapse, *P* primary tumour, *M* metastasis tumour; *B* bone marrow, *T* tumour tissue, *L* lymph node, *C* cytogenetics culture)

^b^Determined by comparing copy-number changes in each chromosome using Pearson’s correlation analysis

### Characterisation of high-risk neuroblastoma PDX models

**Fig. 1:**
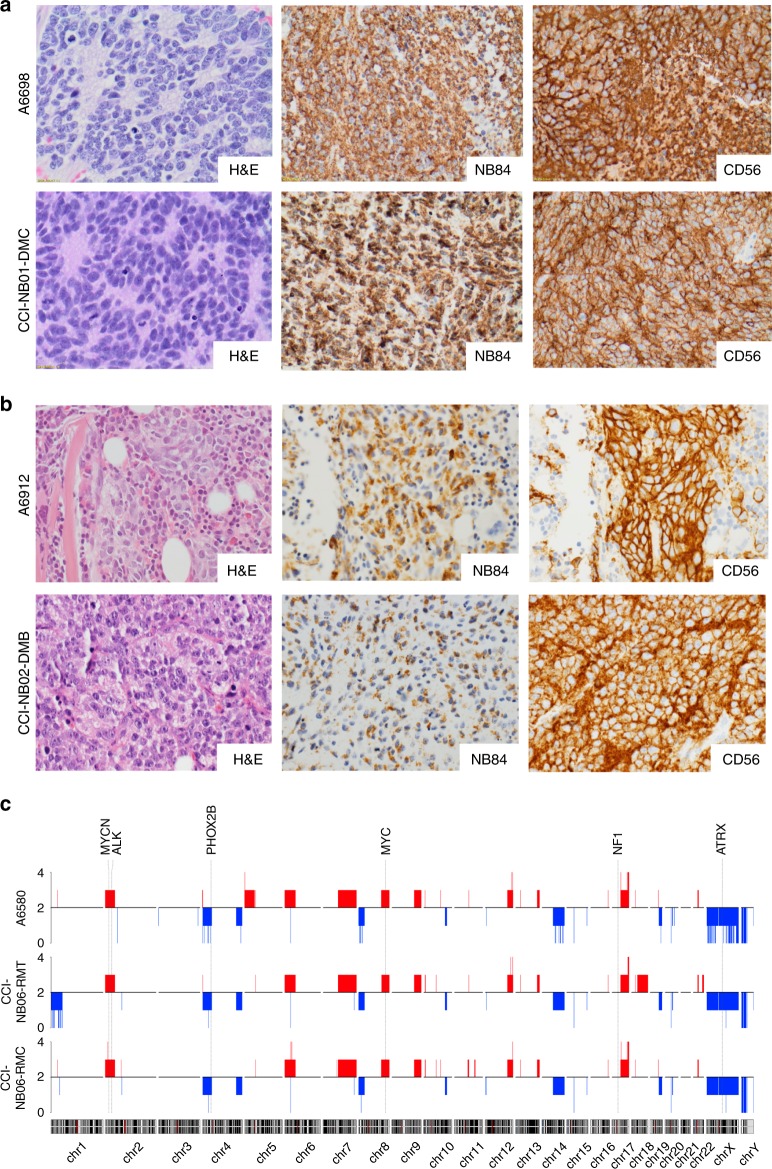
Comparison of patient-derived xenograft models and their originating tumours. **a** Representative morphology comparison between primary tumour specimens and subcutaneous PDX models of patient A6698/CCI-NB01-DMC and **b** patient A6912/CCI-NB02-DMB. In both models, H&E staining shows that cell morphology was maintained during establishment (magnification: ×600). Immunohistochemical staining for markers NB84 and CD56 in PDX tumours recapitulates original patient tumours, confirming the diagnosis of poorly differentiated neuroblastoma (magnification: ×400). **c** Representative comparison of copy-number profiles across the genome in A6580 patient tumour and the corresponding PDX models developed from primary biopsy (CCI-NB06-RMT) and cytogenetic residual cells (CCI-NB06-RMC). Regions of copy-number gain are shown in red and regions of copy-number loss in blue. Chromosomes are arranged sequentially, where chromosomal positions are indicated below, and the relative positions of genes commonly aberrant in high-risk neuroblastoma (MYCN, ALK, PHOX2B, MYC, NF1 and ATRX) are indicated above.

Each of the established PDX models was authenticated as being derived from the donor tumour specimen by STR profiling. In each case, the match was 100% across the 18 amplified loci. Morphologically, all ten established subcutaneous xenografts recapitulated the features of the patient tumours from which they were derived (Supplementary Table S3). Each PDX showed features of poorly differentiated neuroblastoma, being composed of neuroblasts and variable amounts of neuropil while lacking overt ganglionic differentiation and Schwannian stroma (Fig. 1a, b, left panels). On immunohistochemical staining, the xenografts were variably positive for commonly used neuroblastoma diagnostic markers NB84 and CD56 (Fig. 1a, b, centre and right panels), and synaptophysin (not shown). PHOX2B immunohistochemical staining was performed on some xenografts, and with one exception (CCI-NB06-RMC, from A6580) showed nuclear positivity in tumour cells (Supplementary Table S3). Retrospective staining of the A6580 donor tumour, an intracranial metastasis, confirmed the absence of PHOX2B staining.

To determine the extent to which each PDX model maintained the key genetic features of the donor tumour material from which it was derived, high-density SNP array was conducted for each PDX-tumour pair. For the majority of models, copy-number variation in the PDX was highly correlated with that of the patient tumour, as assessed by Pearson’s correlation analysis (Table 1). Correlations were higher for relapse models (*r* = 0.84–0.99) than for diagnosis models (*r* = 0.035–0.82). The PDX models generally conserved the established prognostic molecular markers and recurrent aberrations observed in the original tumour, including chromosome 1p loss and 17q gain, *MYCN* and *ALK* amplification and loss of *PHOX2B*, *TP53*, *NF1* and *ATRX* (Fig. 1c and Supplementary Fig. S1A, B). One diagnosis model (CCI-NB04-DML), derived from patient A7167, was substantially divergent from the donor lymph node metastasis sample (*r* = 0.035), despite confirmation of sample identity by STR profiling (100% match) and histologic features that were consistent with high-risk neuroblastoma. Interrogation of a previous SNP array (Affymetrix Cytoscan 750K array) conducted on this patient sample for clinical care revealed that *MYCN* amplification and the major chromosomal losses (1p, 10q) and gains (1q, 2p, 13q and 17q), were all present in a tumour population that comprised 50% of cells (Supplementary Fig. S1A, C).

Of particular note, two PDX models were established in parallel from patient A6580, the first directly from fresh tumour material (CCI-NB06-RMT) and the second from cytogenetics culture of the fresh tumour material (CCI-NB06-RMC). Compared with the original patient tumour, minor differences of chromosome 1p loss and 18q gain were observed in the PDX derived from fresh tumour material (*r* = 0.88) with even fewer differences in the PDX from cytogenetics culture (*r* = 0.95) (Fig. 1c). The high correlation with the patient material for both models supports the use of residual cells from short-term cytogenetics culture for establishing PDX models for high-risk neuroblastoma.

### Orthotopic inoculation shortens engraftment time and increases engraftment success rate

Despite the high success rate for subcutaneous engraftment of relapse samples, the success rate for diagnosis samples was below 50%, and the relatively long engraftment time poses a barrier to the use of these models in personalised oncology. To determine whether engraftment at alternative anatomical sites could increase engraftment efficiency, tumour cells from three neuroblastoma patients (A6580, A6912 and A7056) were simultaneously inoculated into three cohorts of mice, subcutaneously, intramuscularly or orthotopically (adrenal gland), and monitored for engraftment and tumour growth. In each case, the tumour sample was already known to engraft (Table 1) and was inoculated at 20–100-fold lower cell number than the previously successful subcutaneous engraftment.

**Fig. 2:**
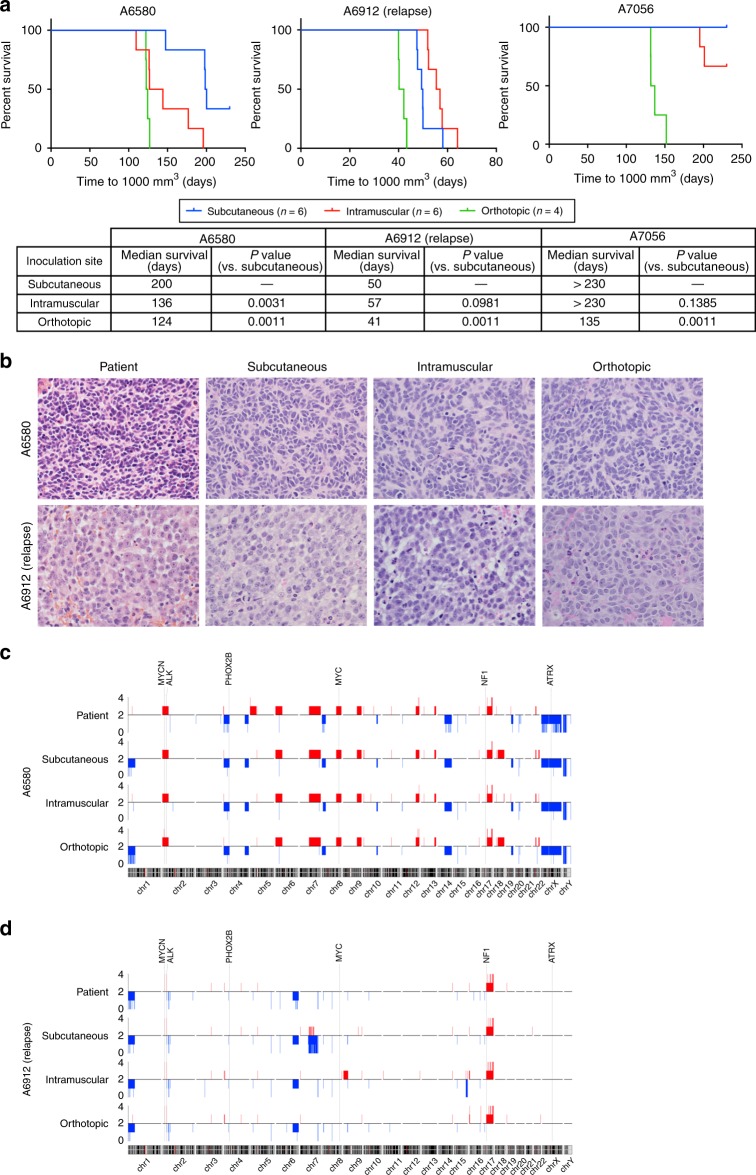
Orthotopic implantation accelerates patient samples engraftment. **a** Kaplan–Meier survival curve of mice engrafted with primary tumour cells from patients A6580, A6912 (relapse) and A7056 at subcutaneous, intramuscular and orthotopic sites. **b** Representative H&E images of patient tumours and the corresponding PDX tumours from the respective inoculation sites. Image magnification: ×600. **c** Copy-number variation analysis for A6580 and **d** A6912 patient samples and PDX models established at each site. Regions of copy-number gain are shown in red and regions of copy-number loss in blue. Chromosomes are arranged sequentially, where chromosomal positions are indicated below, and the relative positions of genes commonly aberrant in high-risk neuroblastoma (MYCN, ALK, PHOX2B, MYC, NF1 and ATRX) are indicated above.

For all three patient tumour samples, orthotopic implantation resulted in the quickest time to measurable engraftment and the shortest median survival time (Supplementary Fig. S2, Fig. 2a). Furthermore, all orthotopically inoculated tumours successfully engrafted, while multiple animals in the intramuscular (A7056) and subcutaneous (A6580 and A7056) groups had no evidence of engraftment or had not reached the event (1000-mm^3^ tumour) after 230 days. Intramuscular engraftment was faster than subcutaneous engraftment in one model (A6580), but slower in another (A6912). To exclude the possibility of bias based on different tumour volume measurement approaches, tumour volumes for all orthotopic xenografts were also obtained at endpoint by using callipers and compared with ultrasound analysis. The two measurement approaches yielded equivalent tumour volumes (Supplementary Fig. S3). The morphologic analysis confirmed that all xenografts exhibited features of poorly differentiated neuroblastoma, consistent with the patient tumours (Fig. 2b). Skeletal muscle was also observed on the xenograft periphery when the tumour cells were inoculated intramuscularly.

To determine whether chromosomal aberrations present in the original tumour are maintained in the PDX models, irrespective of the engraftment site, high-density SNP array was employed. For both patient A6580 (Fig. 2c, Supplementary Table S4) and patient A6912 (Fig. 2d, Supplementary Table S4), xenografted tumours at each site were highly correlated with the patient sample, including conservation of *MYCN* and *ALK* amplification, chromosome 1p loss and chromosome 17q gain in each. Minor differences observed between the A6580 patient sample and the PDX tumours included chromosome 1p loss and 18q gain in the subcutaneous and orthotopic models, while differences observed between the A6912 patient sample and the PDX tumours included a region of copy-number loss in chromosome 7q in the subcutaneous model and a region of copy-number gain in chromosome 9p in the intramuscular model. The orthotopic xenograft for patient A7056 was also highly correlated with the patient sample (Supplementary Fig. 4, Supplementary Table S4). Overall, engraftment site had little impact on tumour copy-number aberrations, with orthotopic and intramuscular xenografts being as representative of the donor tumour as subcutaneous xenografts.

### T-lymphocyte depletion circumvents xenogeneic graft versus host disease

**Fig. 3:**
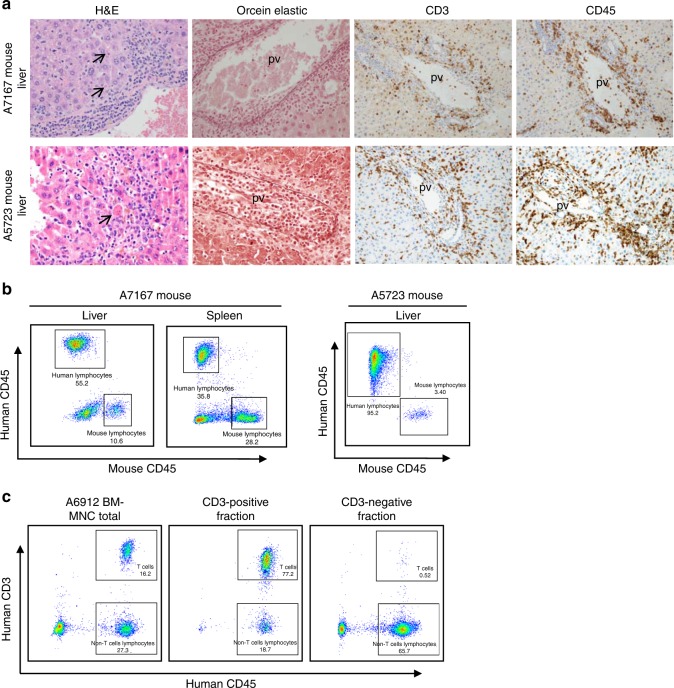
Human lymphocyte infiltration in mouse organs during attempted engraftment of patient samples containing substantial leucocyte population. **a** Histologic appearance of liver from mice inoculated with either lymph node (A7167) or bone marrow (A5723) samples demonstrating mononuclear inflammation of the portal areas, with extension of the inflammatory infiltrate into the hepatic lobule and hepatic injury. The arrow on H&E stain indicates an acidophil body (necrotic hepatocyte). Portal areas are stained with special stain orcein elastic to demonstrate the marked inflammation of portal vein branches. Immunohistochemical staining for human specific lymphocyte antigens CD3 (T lymphocyte) and CD45 (pan-lymphocyte) indicates that the majority of infiltrating mononuclear cells are human T lymphocytes. pv, portal vein branch; bd, interlobular bile duct; ha, hepatic artery branch. Magnification: ×600. **b** Flow cytometry on cells dissociated from mouse liver (A7167 and A5723) and spleen (A7167) demonstrates the presence of CD45-positive human lymphocytes. **c** Human T-lymphocyte depletion of A6912 patient BM-MNC sample at diagnosis prior to successful establishment of the PDX model. The left panel shows total cells population prior to separation with CD3 magnetic beads. Middle and right panels show the cells fraction after separation. Cell population in the negative fraction was used for PDX establishment.

The engraftment of two patient samples containing a substantial lymphocyte population (A7167, lymph node metastasis, and A5723, bone marrow metastasis) led to alopecia and reddening of skin around the ears and eyes in the recipient mice, consistent with xenogeneic graft versus host disease (GvHD). Postmortem histopathological analysis of the liver from mice inoculated with each sample was also consistent with GvHD (Fig. 3a). Mild effacement of liver architecture with expansion of portal tracts by infiltrating activated lymphocytes and occasional neutrophils was observed for both samples. Occasional lymphocytes were seen in close proximity to endothelial cells of the portal vein branches. Expansion of the tunica intima/media of some portal vein branches was seen, accompanied by lymphocytic infiltration of the endothelium (phlebitis) and subjacent connective tissue. The portal lymphocytic infiltrate extended into the adjacent hepatic lobule and was accompanied by necrosis of occasional hepatocytes (acidophil bodies). The mononuclear cell infiltrate was positive for human CD45 and human CD3, confirming these cells as T lymphocytes. Both CD4-positive and CD8-positive T lymphocytes were present (not shown). B lymphocytes were absent from the liver of A7167-inoculated mice based on CD20 staining, but were present in the liver of mice inoculated with material from patient A5723. Staining for the macrophage marker CD68 was negative. Alternative infectious causes of liver injury were excluded by testing of the animal facility for *Helicobacter* or norovirus infection and the absence of *Helicobacter* organisms in the hepatic tissue. There was no evidence of metastatic neuroblastoma to the liver in either case.

Flow cytometric analysis of mononuclear cells isolated from liver and spleen of the mice indicated 55% and 36% CD45-positive human leucocytes in the A7167 mouse liver and spleen, respectively, and 95% CD45-positive human leucocytes in the A5723 mouse liver (Fig. 3b). To preclude the development of xenogeneic GvHD, in subsequent engraftment experiments, patient lymph node or bone marrow samples were subjected to magnetic bead separation to deplete CD3-positive human T lymphocytes prior to engraftment. For patient A6912 (bone marrow metastatic disease), human T lymphocytes were reduced from 16% to less than 1% in post separation (Fig. 3c). This sample subsequently engrafted within 96 days (Table 1, CCI-NB02-DMB), with no signs of xenogeneic GvHD.

### Development of EBV-associated atypical B-lymphoid proliferation from a pleural fluid sample

**Fig. 4:**
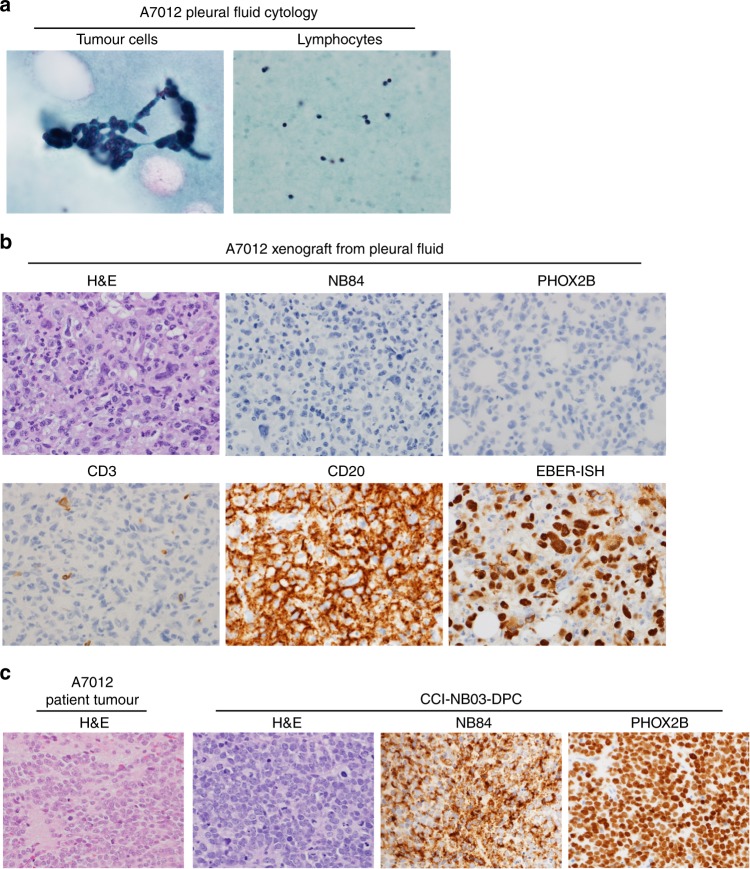
Lymphomatous proliferation during the attempted establishment of a high-risk neuroblastoma PDX model. **a** Cytology smear preparation of pleural fluid sample taken from patient A7012 shows the presence of metastatic neuroblastoma cells accompanied by background lymphocytes (Papanicolaou staining). **b** Morphologic appearance of A7012 xenograft tumour derived from pleural fluid. H&E staining shows a tumour composed of large variably pleomorphic cells. On immunohistochemical staining, the xenograft is positive for CD20 and negative for CD3, NB84 and PHOX2B. EBV-encoded RNA in situ hybridisation (EBER-ISH) confirms the presence of EBV within tumour cells. Magnification: ×600. **c** Successfully established A7012 PDX model (CCI-NB03-DPC) from cytogenetic residual cells shows similar morphologic appearance with patient tumour (H&E staining). Immunohistochemical staining of the xenograft shows positivity for NB84 and PHOX2B, confirming the diagnosis of poorly differentiated neuroblastoma. Magnification: ×600.

An attempt to generate a xenograft model from a pleural fluid specimen (patient A7012) containing both metastatic neuroblastoma cells and inflammatory cells, including small lymphocytes and neutrophils (Fig. 4a), produced a tumour mass that failed to recapitulate human neuroblastoma. This xenograft comprised an infiltrate of large cells with variably atypical nuclei and ample vacuolated to the eosinophilic cytoplasm. An accompanying patchy inflammatory cell infiltrate was also seen, including apparent lymphocytes and neutrophils. This xenograft was positive for the B-lineage marker CD20 and negative for the neuroblastoma markers PHOX2B and NB84, and negative for the T-cell marker CD3 (Fig. 4b). Additional flow cytometry analysis demonstrated the presence of a CD19-positive B-lymphocyte population (Supplementary Fig. S5); however, clonality studies were not performed. Strong nuclear positivity for EBER-ISH staining (Fig. 4b) and CD20 positivity confirms this tumour as an EBV-associated atypical B-lymphoid proliferation. In contrast, a PDX model established for patient A7012 from cytogenetic residual cells (CCI-NB03-DPC) showed features of poorly differentiated neuroblastoma consistent with the original tumour, NB84 and PHOX2B positivity (Fig. 4c), and strongly correlated with the patient material by SNP array (Supplementary Fig. S1, *r* = 0.71).

### Comparative high-throughput drug screening of neuroblastoma patients and PDX-derived cells

**Fig. 5:**
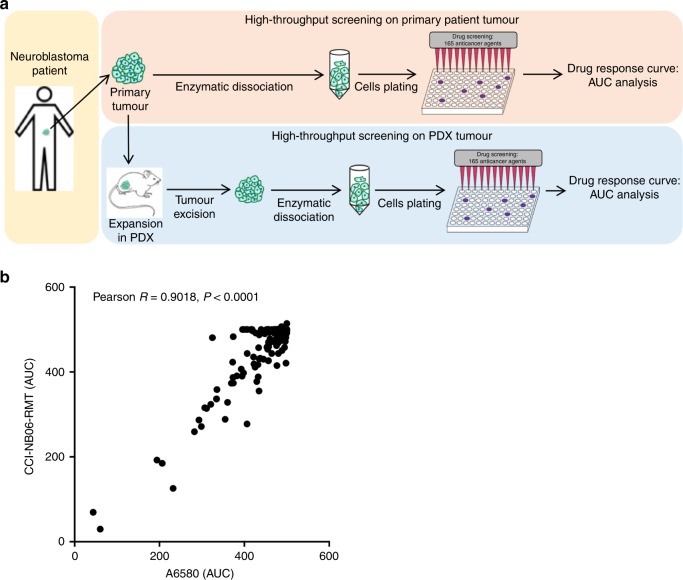
High-throughput screening of patient tumour cells and PDX cells. **a** Schematic diagram of sample processing for drug screening. Primary patient tumour is dissociated into a single-cell suspension and then plated into 384-well plates. After 24 h, cells were treated with 165 anticancer agents and then incubated for 72 h. Cell-titer glo is used to indicate cell viability and generate drug response curves for AUC analysis. In the case of limited patient material, tumour has to be expanded in PDX model prior to drug screening. PDX tumour is then processed in the same way as the patient tumour for drug screening. **b** Drug sensitivity in patient cells (A6580) and PDX cells (CCI-NB06-RMT) shows significant correlation based on AUC analysis (*R* = 0.9018, *P* < 0.0001).

To further assess the biological similarly of primary patient cells and those expanded via mouse engraftment, a high-throughput screen of 165 anticancer drugs was performed on dissociated cells from both the A6580 patient sample and the first passage of the cognate PDX (CCI-NB06-RMT) under identical conditions (Fig. 5a). Prior to screening, the PDX cells were analysed for GD2 positivity by flow cytometry to determine neuroblastoma cell content and were found to be 96.6% GD2-positive (Supplementary Fig. S6). The drug library consists of clinically approved chemotherapies and targeted agents, and also experimental anticancer agents currently under investigation. Overall, cells derived from both the initial patient material and PDXs showed highly similar sensitivity to chemotherapeutic agents. The AUC values for all tested drugs in patient cells were highly correlated with those in the PDX cells (*r* = 0.9018, *P* < 0.0001, Fig. 5b), suggesting that neuroblastoma tumour cells expanded as xenografts may be fit-for-purpose for personalised drug sensitivity testing.

## Discussion

PDX models are known to preserve many of the key biological properties of the tumours from which they were derived and remain stable across passages.^11,27^ These models are predictive of clinical outcomes and therefore are an excellent platform for preclinical drug testing and as avatars for personalised medicine.^8,9,11^ This study extends previous neuroblastoma PDX model establishment reports^19–28^ by demonstrating that PDX models can also be developed from diagnostic cytogenetic culture without additional loss of fidelity, that more rapid engraftment can be achieved with orthoptic implantation and that additional sample processing can circumvent loss of models by xenogeneic GvHD.

Our subcutaneous engraftment success rates of 44% (4 of 9) for diagnosis patients and 100% (5 of 5) for relapse patients compare favourably with a recent report^26^ in which neuroblastoma samples were engrafted para-adrenally with a success rate of 24% at diagnosis (9 of 39) and 33% at relapse (1 of 3). Several factors might contribute to the variation of engraftment rate between these studies, including the source of patient material, time to engraftment, number of cells inoculated and engraftment technique. In an earlier study,^21^ the subcutaneous engraftment rate of PDX from neuroblastoma patients at the time of diagnosis was 34% (20 of 58) for all patients attempted, and 45% (15 of 33) from INSS stage IV disease, which is similar to the current study. We found that histologic and molecular features are generally preserved in high-risk neuroblastoma PDX models commensurate with previous reports.^19–28^ The high success rate for samples from cytogenetics culture by comparison with samples from other patient sources that failed to engraft, strongly suggests that these materials should be accessed for model development where possible. While one PDX model (CCI-NB04-DML from A7167) developed from a lymph node metastasis via cytogenetics cells showed very poor correlation with the originating tumour material based on the CNV profile, further analysis of a clinical SNP array for these samples suggests clonal selection in the PDX, and good concordance with the expanded clone in the original patient material. More generally, evolution and copy-number alteration that occurs during development of PDX models might provide explanation for other differences.^34,35^

With further optimisation, other potential samples, such as pleural fluid and lymph node metastasis biopsy, can be used as valuable resources for PDX development, increasing engraftment success rate per patient, an essential advance for implementing personalised treatment in this childhood malignancy. Avoiding the loss of engrafted samples due to mouse pathologies is also critical for reliable model development and judicious choice of recipient mice and sample-processing approaches is essential. Highly immune-deficient NSG mice are advantageous for tumour engraftment, have significantly longer life spans than other *scid* mutant strains and do not develop spontaneous thymoma,^36^ which are important considerations when engraftment time is long, as is frequently the case for neuroblastoma models. While the absence of functional NK cells renders NSG mice highly susceptible to xenogeneic GvHD development,^37^ we show that this can be avoided by T-lymphocyte depletion of the patient sample.

Development of EBV-associated atypical B-lymphoma proliferation or EBV-related B-cell lymphoma has been previously described after attempted xenografting of other human tumour types in immune-deficient mice^30,38–43^ and the incidence rate in NSG mice has been reported to be as high as 32%.^41^ In healthy individuals, EBV infection is benign with reactivation and subsequent proliferation of EBV-infected lymphocytes suppressed by immune detection and response,^44^ whereas in an immunodeficient patient, EBV can transform B cells into the growth phase and trigger the lymphoproliferative disease.^45^ Similarly, the lack of immunosurveillance in recipient mice allows the proliferation of the EBV-infected lymphocytes contained in the patient.^38–42^ PCR-based detection of the EBV BamHI W region, a major internal repeat in EBV genome, in the patient specimen to be engrafted, has been reported to correlate with EBV-related lymphoid proliferation in the derivative PDX and may allow identification of samples requiring lymphocyte depletion.^44^ Alternatively, atypical lymphoid proliferations might be obviated by administration of rituximab, an anti-CD20 antibody, as the rate of lymphoma incidence in ovarian cancer PDX models has been reported to decline from 11.1 to 1.88% after the addition of rituximab.^46^ Aside from prevention, a robust approach to identify atypical lymphoid proliferation is essential to maintaining xenograft validity and mandatory prior to additional clinical assays, such as drug screening, being performed on the xenograft material.

A major challenge for the use of high-risk neuroblastoma PDX models in personalised medicine is the development, validation and application of models in a timeframe that can still inform individual patient treatment. Approaches that shorten engraftment time or increase engraftment success rates without compromising model fidelity are essential to make this approach broadly applicable. Our observation that for each of three patient samples, orthotopic adrenal engraftment was substantially faster than either subcutaneous or intramuscular engraftment suggests one approach to reducing engraftment time. The reasons for more rapid adrenal engraftment are not clear; however, the adrenal gland is the most common site of origin for primary neuroblastoma. Alternatively, previous studies have suggested that non-subcutaneous sites may provide greater protection of the engrafted cells against hypothermic insult,^30^ or a greater capacity for angiogenesis.^22,25,31^ While enabling a shorter engraftment time, orthotopic inoculation is more technically challenging than subcutaneous inoculation and animal monitoring typically requires in vivo imaging,^47,48^ which can be particularly labour intensive for the frequent monitoring required in preclinical testing.^32,48^ Our observations that orthotopic, intramuscular and subcutaneous xenografts are equally representative of the donor tumour suggest that engraftment approaches can be selected based on utility.

In addition to in vivo preclinical testing, limited patient material expanded as a PDX model can be used as a primary short-term culture for high-throughput ex vivo testing of drug sensitivity, including for combination chemotherapy.^49^ This study demonstrated that PDX-derived cells were dependable alternatives for screening high-risk neuroblastoma, with ex vivo drug sensitivity of primary patient cells and PDX-expanded material being highly correlated.

In summary, we identify dependable engraftment approaches that increase the likelihood of developing informative preclinical models for individual high-risk neuroblastoma patients within a clinically relevant timeframe and reliably expand limited patient material for ex vivo and in vivo drug testing.

## Supplementary information

Supplementary materials

## Data Availability

The data that support the findings of this study are available from the corresponding author upon reasonable request.
