# Supplementary material for: Accelerating development of high-risk neuroblastoma patient-derived xenograft models for preclinical testing and personalised therapy
Source: Br J Cancer. 2020 Jan 10;122(5):680–91. doi: 10.1038/s41416-019-0682-4 (PMC7054410; doi:10.1038/s41416-019-0682-4)
Supplement: Supplementary file 1 — Supplementary materials [file 41416_2019_682_MOESM1_ESM.docx]

**Supplementary tables**

**Table S1.** Clinical outcome of neuroblastoma patient with sample attempted for PDX establishment at diagnosis

| **Engraftment outcome** | **Number of deceased patients** | **Number of alive patients** |
| --- | --- | --- |
| Successful | 3 | 1 |
| Failed | 0 | 5 |

Engraftment was associated with poor outcome*

**P*=0.048, Fisher’s exact test

**Table S2.** Engraftment time of established neuroblastoma PDX model at secondary passage

| **PDX model** | **Phase of therapy** | **Mean time to endpoint* (days)** | **Number of cells inoculated per mouse (x 10^6^)** |
| --- | --- | --- | --- |
| CCI-NB01-DMC | diagnosis | 59 | 10 |
| CCI-NB02-DMB | diagnosis | 49 | 10 |
| CCI-NB03-DPC | diagnosis | 56 | 10 |
| CCI-NB04-DML | diagnosis | 53 | 5 |
| CCI-NB05-RMB | relapse | 157 | 10 |
| CCI-NB06-RMT | relapse | 281 | 1 |
| CCI-NB01-RMT | relapse | 77 | 10 |
| CCI-NB02-RPT | relapse | 35 | 8 |
| CCI-NB07-RMT | relapse | 210 | 10 |

*Endpoint = tumour size equal to 1000 mm^3^

| **Table S3.** Summary of histopathological findings for patients and corresponding xenografts | | | | | | | | | | | | |  |  |
| --- | --- | --- | --- | --- | --- | --- | --- | --- | --- | --- | --- | --- | --- | --- |
| **Patient** | **Phase of therapy** | **primary tumor pathology** | **Patient tumour immunohistochemistry** | | | | **PDX ID** | **Xenograft tumour pathology** | **Xenograft tumour immunohistochemistry** | | | | | |
|  |  |  | **NB84** | **CD56** | **Synapto-physin** | **PHOX2B** |  |  | **NB84** | **CD56** | **Synapto-physin** | **PHOX2B** | | |
| A6698 | Diagnosis | Poorly-differentiated NB | Positive | Positive | Positive | n/a | CCI-NB01-DMC | Poorly-differentiated NB | Positive | Positive | Positive | n/a | | |
| A6912 | Diagnosis | Undifferentiated NB | Positive | Positive | Positive | n/a | CCI-NB02-DMB | Poorly-differentiated NB | Positive | Positive | Positive | n/a | | |
| A7012 | Diagnosis | Poorly-differentiated NB | Positive | Positive | Positive | n/a | CCI-NB03-DPC | Poorly-differentiated NB | Positive | n/a | n/a | Positive | | |
|  |  |  |  |  |  |  | Growth from pleural fluid | EBV-associated B-lymphoid proliferation | Negative | Negative | Negative | Negative | | |
| A7167 | Diagnosis | Poorly-differentiated NB | Positive | Positive | Positive | n/a | CCI-NB04-DML | Poorly-differentiated NB | n/a | n/a | Positive | Positive | | |
| A5723 | Relapse | Poorly-differentiated NB | Positive | Positive | Positive | n/a | CCI-NB05-RMB | Poorly-differentiated NB | Positive | Positive | Positive | n/a | | |
| A6580 | Relapse | Poorly-differentiated NB | n/a | Positive | Positive | Negative | CCI-NB06-RMT | Poorly-differentiated NB | Positive | Positive | Positive | n/a | | |
|  |  |  |  |  |  |  | CCI-NB06-RMC | Poorly-differentiated NB | Positive | Positive | Negative | Negative | | |
| A6698 | Relapse | Poorly-differentiated NB | Positive | Positive | Positive | n/a | CCI-NB01-RMT | Poorly-differentiated NB | Positive | Positive | Positive | n/a | | |
| A6912 | Relapse | Undifferentiated NB | Positive | Positive | Positive | n/a | CCI-NB02-RPT | Poorly-differentiated NB | n/a | n/a | Positive | Positive | | |
| A7056 | Relapse | Poorly-differentiated NB | Positive | n/a | Positive | n/a | CCI-NB07-RMT | Poorly-differentiated NB | Positive | n/a | n/a | Positive | | |
| NB: neuroblastoma, EBV: Epstein Barr virus, n/a: IHC not performed | | | | | | | | | | | | | |  |

**Table S4.** Correlation of PDX models established at each anatomical site with the donor tumour

| **Patient ID** | **Engraftment site** | **Correlation of PDX with patient tumour (r value)^1^** |
| --- | --- | --- |
| A6580 | subcutaneous | 0.88 |
| A6580 | orthotopic | 0.88 |
| A6580 | intramuscular | 0.95 |
| A6912 | subcutaneous | 0.82 |
| A6912 | orthotopic | 0.99 |
| A6912 | intramuscular | 0.82 |
| A7056 | orthotopic | 0.83 |

^1^Determined by comparing copy number changes in each chromosome using Pearson’s correlation analysis.

**Supplementary figures**

**Figure S1.** Copy number profiles across the genome in the PDX and its originating patient tumour material. **(A)** Regions of copy number gain are shown in red and regions of copy number loss in blue. Chromosome are arranged sequentially, where chromosomal positions are indicated below, and the relative positions of genes commonly aberrant in high-risk neuroblastoma (MYCN, ALK, PHOX2B, MYC, NF1, ATRX) are indicated above. A7167 panel includes an additional copy number analysis conducted as part of clinical care using Affymetrix Cytoscan (750K SNP loci read). All other patients and PDXs SNP array data were obtained using the higher resolution Illumina InfiniumOmni2.5 (2.5M SNP loci read). **(B)** SNP data at higher resolution for chromosome 6 for A6912 tumour at diagnosis. **(C)** B allele frequency and Log R ratio of A7167 patient for copy number analysis using Affymetrix Cytoscan.

**Figure S2.** Tumour growth curves for mice engrafted with primary tumour cells from patients A6580, A6912 (relapse), and A7056 at subcutaneous, intramuscular, and orthotopic sites.

**Figure S3.** Comparison of tumour measurement obtained by ultrasound and caliper. Mice bearing orthotopic tumours were euthanised once the size reached 1000 mm^3^, as measured by ultrasound. Tumour volume from each animal was subsequently measured *ex vivo* by caliper for comparison. Data point with same colour represents tumour from one animal (*P=*0.6023, n.s.).

**Figure S4.** Copy number profiles across the genome of orthotopically engrafted A7056 xenograft and its originating patient tumour material. Regions of copy number gain are shown in red and regions of copy number loss in blue. Chromosome are arranged sequentially, where chromosomal positions are indicated below, and the relative positions of genes commonly aberrant in high-risk neuroblastoma (MYCN, ALK, PHOX2B, MYC, NF1, ATRX) are indicated above.

**Figure S5.** Flow cytometry on dissociated tumour cells with antibody against CD19 confirmed the presence of B-lymphocytes.

**Figure S6.** Human neuroblastoma cell content in dissociated CCI-NB06-RMT PDX tumour for high-throughput screening.
